# A combined 3D printing/CNC micro-milling method to fabricate a large-scale microfluidic device with the small size 3D architectures: an application for tumor spheroid production

**DOI:** 10.1038/s41598-020-79015-5

**Published:** 2020-12-17

**Authors:** Ebrahim Behroodi, Hamid Latifi, Zeinab Bagheri, Esra Ermis, Shabnam Roshani, Mohammadreza Salehi Moghaddam

**Affiliations:** 1 Laser and Plasma Research Institute, Shahid Beheshti University, 1983963113 Tehran, Iran; 2 Department of Physics, Shahid Beheshti University, 1983963113 Tehran, Iran; 3 Faculty of Life Sciences and Biotechnology, Shahid Beheshti University, 1983963113 Tehran, Iran

**Keywords:** Lab-on-a-chip, Cancer models, Engineering, Optics and photonics

## Abstract

The fabrication of a large-scale microfluidic mold with 3D microstructures for manufacturing of the conical microwell chip using a combined projection micro-stereolithography (PµSL) 3D printing/CNC micro-milling method for tumor spheroid formation is presented. The PµSL technique is known as the most promising method of manufacturing microfluidic chips due to the possibility of creating complex three-dimensional microstructures with high resolution in the range of several micrometers. The purpose of applying the proposed method is to investigate the influence of microwell depths on the formation of tumor spheroids. In the conventional methods, the construction of three-dimensional microstructures and multi-height chips is difficult, time-consuming, and is performed using a multi-step lithography process. Microwell depth is an essential parameter for microwell design since it directly affects the shear stress of the fluid flow and the diffusion of nutrients, respiratory gases, and growth factors. In this study, a chip was made with microwells of different depth varying from 100 to 500 µm. The mold of the microwell section is printed by the lab-made PµSL printer with 6 and 1 µm lateral and vertical resolutions. Other parts of the mold, such as the main chamber and micro-channels, were manufactured using the CNC micro-milling method. Finally, different parts of the master mold were assembled and used for PDMS casting. The proposed technique drastically simplifies the fabrication and rapid prototyping of large-scale microfluidic devices with high-resolution microstructures by combining 3D printing with the CNC micro-milling method.

## Introduction

3D printing technology is becoming a promising approach in the production of microfluidic devices due to its amazing advantages over traditional methods, particularly mask-based photolithography^1,2^. Cost-effectiveness, multiple thickness generation, 3D architecture creation, design simplicity, and elimination of the need for cleanroom facilities are key advantages of using 3D printers in microfluidic device manufacturing^3^. The 3D printer is used in the manufacturing process of a microfluidic device in two ways. Fabricating the entire microfluidic device or making a mold, which in the next step is used for casting with various polymers^4^. Making the mold using the 3D printer helps to obtain polydimethylsiloxane (PDMS) based microfluidic devices^5^.

PDMS has become a successful polymeric substrate material for rapid prototyping owing to its low cost, ease of fabrication, gas permeability, optical transparency, multi-substrate adhesion, chemical inertness, biocompatibility, and ability to form any geometry^6^. Due to PDMS's surface characteristics, it necessary to coat the surface of the 3D printed template before PDMS casting. It turns out that the PDMS layer on the surface of the printed component does not cure or cannot peel off after curing, probably because of the remaining monomers and catalysts on the surface of printed mold^7^. The coating layer provides a barrier to the 3D printed material so that the PDMS polymerization is carried out on the surface of the mold, and the PDMS layer can be easily peeled off^4^.

Another problem with using a 3D printer in manufacturing microfluidic devices is to achieve the high resolution of construction that many microfluidic applications require. The resolution depends on both the mechanical control of the printer and the printing materials^8^. Among the various 3D printing technologies, micro-stereolithography (µSL) and projection micro-stereolithography (PµSL) approaches usually offer the smoothest surfaces and the best resolution at a reasonable price^4^. In μSL technology, a UV laser scanning technique and photopolymerization process are used to create a three-dimensional microstructure. In this method, 3D microstructures are created additively, layer by layer, according to the laser scan pattern^9,10^. In PμSL technology, the laser scanner is replaced by a spatial light modulator as a dynamic mask generator^11^. Printing speed is the main advantage of the P*µ*SL over the *µ*SL^10^.

Usually, the achievement of the high resolution in 3D printing limits the printing size, which is another challenge in this field^12^. Since most microfluidic chips consist of channel systems, chambers, and various control and management elements, it is expected that the final chip size often increases on a centimeter scale. Therefore, an ideal method for making microfluidic devices should be able to create microstructures with appropriate resolution and to have the flexibility to make an object of different sizes. For example, Waldbaur et al. used a high resolution DMD-based 3D printer to print a master mold of a tree-based gradient generator chip. In this regard, they solved the mentioned problem by translating the projected images; consequently, they printed a large layout (3 × 4 cm) with proper resolution^13^. The complexity of creating an object with multiple thicknesses, time-consuming, and low resolution on stitching surfaces were the limitations of their approach. Also, Bryan D. Moran has patented a large area projection micro stereolithography (LAPuSL) system that uses an addressable spatial light modulator (SLM) in coordination with an optical scanning system to make large 3D objects^14^. Zheng et al. used this method to produce hierarchical metamaterials with disparate three-dimensional features^15^. This technique can be used as an alternative method that could allow an entire high-resolution microfluidic mold to be printed at once.

In the present study, we have developed a new process for the fabrication of a large size master mold containing high-resolution 3D microstructures. Parts of the mold with microstructure features were fabricated by the lab-made PµSL 3D printer^16^ with 6 and 1 µm lateral and vertical resolutions, respectively. Other parts of the mold, such as the main chamber and microchannels, were manufactured using the CNC micro-milling method. Additionally, we used PMMA/chloroform solution for surface treatment of 3D printed mold before PDMS casting. With this treatment, mold surface smoothness improved, PDMS was cured on the mold surface and easily peeled off after curing, and also, the mold can be reused several times.

To confirm this approach and highlight the flexibility of the proposed method, we made a microwell-based microfluidic chip for tumor spheroid formation. It has been shown that cancer cell spheroids imitate in-vivo tumor microenvironments owing to the similarity in cell–cell and cell–matrix interaction and are therefore appropriate for in-vitro drug development and evaluation processes^17–19^. Among the various methods, microwell chips have become a valuable tool for effective spheroid formation, since the sample and reagent volume is minimized, the cell microenvironment is precisely manipulated, the spheroid size is adequately controlled, and the operation is simplified^20–22^. In this type of microfluidic device, cells form aggregates in the microwells and increase intercellular contacts, resulting in increased E-cadherin expression. These E-cadherin interactions can result in the formation of the compact structures called spheroids^23^.

However, there are several commercial multi-well microplate platforms for making spheroids, such as InSphero Gravity TRAP (InSphero, Switzerland), SPHERICALPLATE 5D (Kugelmeier), and AggreWell (STEMCELL Technologies). These products offer a convenient and standard approach to the high-throughput production of uniformly sized spheroids. But there are generally several limitations to the use of these products. For example, AggreWell plates are limited in changing the medium during pipetting, which leads to disruption of the spheroids within the microwells, consequently complicates the long-term cell culturing and drug treatment applications^24,25^. Besides, the depth and diameter of microwells in commercial microplates are fixed; thus, the spheroid's size can only be controlled by the cell density^24^. Finally, the price of these commercially available disposable plates is very high, which can be a limiting factor, especially when more extensive experiments are required^26^. However, the microfluidic nature of spheroid production and storage conditions provided high precision control of the medium exchange and drug treatment.

Various groups investigate the influence of different variables such as the shape, size, and distribution of microwell on the spheroid formation^27–31^. In Table 1, important parameters of microwells that are effective in the spheroid generation are demonstrated. Furthermore, in the last two columns of this table, 3D printing and lithography methods are compared to fabricate the desired parameter for master mold manufacturing. Since microwell depth is an essential parameter for microwell design, which directly affects the shear stress of fluid flow and diffusion of the reagents, the chip was developed with microwells of various depths to investigate the effect of depth on spheroid formation^32,33^. In the conventional method of manufacturing microfluidic chips, accurate and repeatable height adjustment is particularly difficult, especially at a height greater than 200 micrometers^7,34^. Also, creating multiple heights in one chip is not easy^9,35^. In this study, a conical microwell chip with variable depths was developed. The mold of the microwell section was fabricated using high resolution lab-made PµSL 3D printer. The conical shape at the bottom of the microwell contributes to better cell aggregation, which leads to an efficiency of spheroid formation^27,32,33^. Notably, the resolution of the printer can provide the required curvature at the desired scale. Finally, the glioblastoma cell line was applied to demonstrate the application of the proposed chip, and the optimal depth was determined for the best formation condition of spheroids.

**Table 1:** Important parameters of microwells that are effective in the spheroid generation.

Parameter	Properties	Notes	3D printed fabrication	Conventional lithography fabrication
Microwell shape (cross-section)	Square, hexagonal, circular and triangular	Some reports showed that hexagonal and circular shapes are better than others to form founded spheroid^6,27,36,37^	✓	✓
Microwell diameter	100–800 µm	Some reports evaluate the well diameter on spheroid size^27,38–40^	Less than 200 µm performed only with a high-resolution 3D printer^2–4,41^	✓
Microwell depth	100–1000 µm	The appropriate depth for making a spheroid of the desired size is determined based on the diameter^24,27,38,39^	✓	The difficulty for fabrication depth more than 200 µm^7,29^
Microwell bottom	V-conical, V-spindle, U-shape	Help to cell aggregation, form a single and rounded spheroid^29,30,37,42,43^	Only with a high-resolution 3D printer^2–4,41^	×
	Flat-bottom	Simple fabrication^40,44^	✓	✓
Microwell type	Decreasing the cross-section with increasing the depth (conical, spindle, reverse pyramidal)	Help to cell aggregation, form a single and rounded spheroid^24,28,45,46^	Only with a high-resolution 3D printer^2–4,41^	×
	Fixed cross-section (cylindrical, hexagonal prism, and cubic)	Simple fabrication^40,44^	✓	✓

## Materials and methods

### Chemicals

Phosphine oxide (Irgacure 819) 1-phenylazo-2-naphthol (Sudan I), 1,6-hexanediol diacrylate (HDDA), and phenylbis(2,4,6-trimethylbenzoyl) were purchased from Sigma Aldrich company. Isopropanol was obtained from Mojalali corporation (Iran, Tehran). PMMA powder and chloroform were purchased from Merck company. PDMS (Sylgard 184 Elastomer Kit, Dow Corning, Midland, MI, USA) was obtained from Dow corning company. PMMA sheet was bought from Best company (Taiwan). Fetal bovine serum (FBS), Dulbecco's modified Eagle's medium (DMEM) were obtained from Gibco (Grand Island, NY, USA). Phosphate buffered saline (PBS), penicillin/streptomycin, trypsin/EDTA were purchased from Merck company. Propidium iodide (PI) and Acridine Orange were purchased from Sigma Aldrich company.

### Design of the microfluidic device

Different parts of the master mold were designed using SOLIDWORKS 2016 software (Dassault Systems, France). The chip consists of 35 microwells, which are divided into five groups of 7. The microwells are placed in a chamber that has one inlet and one outlet. The inlet and outlet are connected to the chamber with channels of 800 μm width and 6000 μm length. The height of 200 μm was taken into account for the chamber. The microwells are designed to be conical in the axial direction with an angle of 25°, and the upper plane is a regular hexagon with a side length of 300 μm. This shape reduces the dead area of walls between the microwells and subsequently decrease the cell loss. In this study, two chips were designed and tested. In Chip 1, the depth of each group of seven microwells was changed from 100 to 500 μm in increments of 100 μm to investigate the effect of depth on the cell trapping and the spheroid formation. In Chip 2, according to the results of Chip 1, the depth of all microwells was 300 μm.

Due to the complexities of the microwell part, the manufacturing process for the microwell section of the master mold carried out using a lab-made PµSL 3D printer^16^. Since the lateral resolution of the 3D printer is about 6 µm, the maximum size of the printed object is 7.7 mm × 4.8 mm, which is too small to make a chip. Therefore, the entire master mold cannot be built by the 3D printer itself. For this reason, other parts of the chip, such as microchannels and the main body of the chip, were manufactured using CNC micro-milling. In the next step, the different components are assembled to fabricate the final master mold.

**Figure 1:**
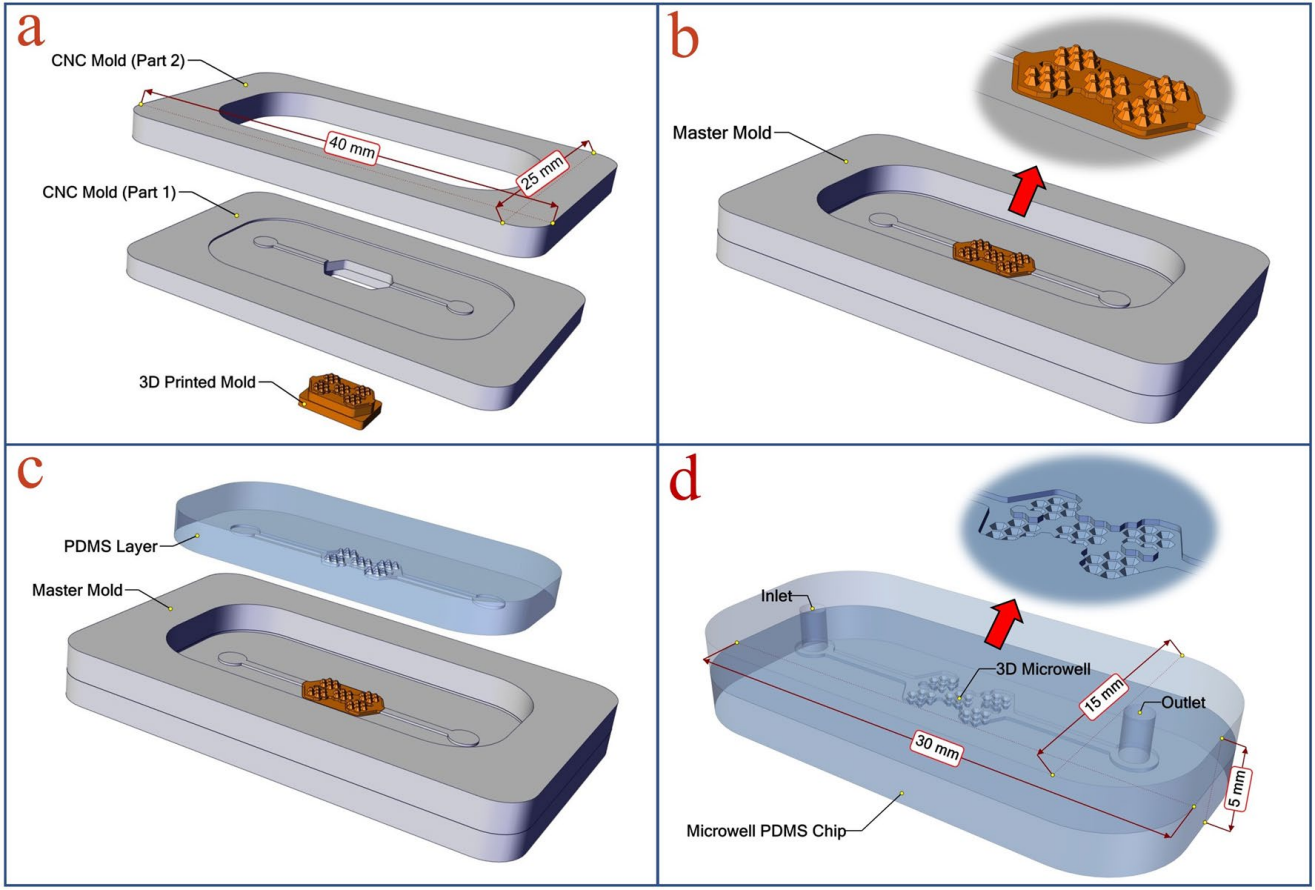
The manufacturing steps of the master mold: (**a**) the exploded view of the master mold that consists of one 3D printed mold and two CNC micro-milling parts. (**b**) The assembled master mold. (**c**) The PDMS casting process. (**d**) The final PDMS microfluidic chip. [This figure was created by SOLIDWORKS 2016 (www.solidworks.com)].

Figure 1 illustrates the manufacturing steps of the master mold. Figure 1a shows different parts of the master mold, consisting of one 3D printed template and two CNC micro-milling parts, in an exploded view. In CNC part 1, the channels and the position of the 3D printed mold are created. The CNC part 2 provides the chamber required to insert the PDMS polymer. Figure 1b schematically shows the final assembled master mold. CNC micro-milling parts are bonded to each other by chloroform via the solvent bonding method. The 3D printed template is located in the empty part of the center of the CNC part 1. It is then fixed in its place by reusable and removable adhesive putty (UHU Patafix). After using the master mold, the 3D printed template can be easily separated from the CNC micro-milling parts and be replaced with another if necessary. Figure 1c illustrates the PDMS casting process. Figure 1d shows the final PDMS microfluidic chip. The chip is 30 × 15 × 5 mm and consists of two PDMS parts that are bonded to each other by the plasma bonding process. The lower part contains microwells, chambers, and channels. The upper part is the chip cover, and the inlet and outlet holes are punched therein.

### Flow simulation

For investigating the velocity distribution inside the chamber and microwells, the series of finite-element method (FEM) simulations were carried out using COMSOL Multiphysics software. The governing equations to simulate the Newtonian fluids inside the chip are continuity equation (Eq. 1), and three dimensional (3D) Navier–Stokes equation (Eq. 2)

1 $$\nabla \cdot V = 0$$

2 $$\rho \left( {\frac{\partial V}{{\partial t}} + V \cdot \nabla V} \right) = - \nabla p + \mu \nabla^{2} V$$

where V, ρ, p, and µ denote velocity vector, density, pressure, and viscosity, respectively. The Eq. (2) is simplified by neglecting the effect of body force. Boundary conditions in this simulation were Steady-state, incompressible flow, and no-slip wall condition. The flow rate at the inlet was assumed 50 μL/min, and zero pressure was specified at the outlet. Also, for the discretization of the computational domain, tetrahedral grids were implemented^47^.

### 3D printing of microwell mold

**Figure 2:**
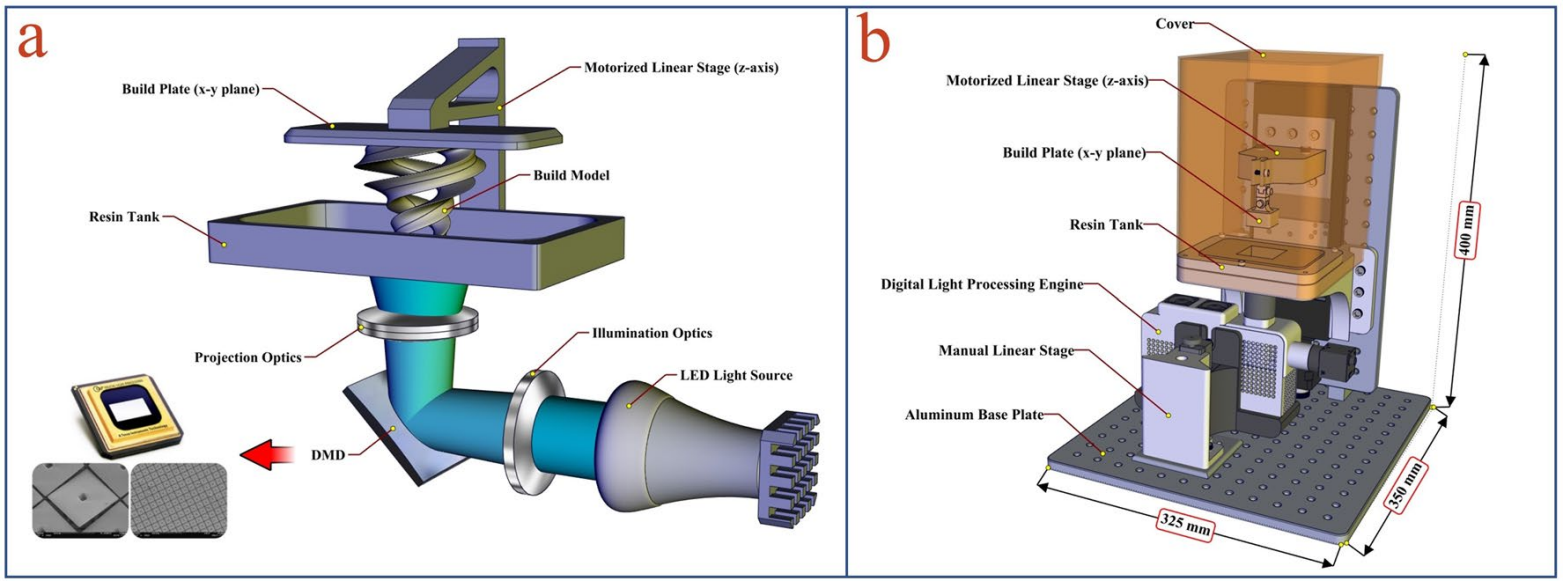
(**a**) The schematic illustration of the bottom-up configuration and (**b**) The 3D CAD model of the designed compact PµSL 3D printer. [This figure was created by SOLIDWORKS 2016 (www.solidworks.com)].

For producing the 3D printed mold, the lab-made PμSL 3D printer was used. The process of making this 3D printer previously reported^16^. In the PμSL approach, a 3D microstructure is built using a UV light projection technique and photopolymerization process. In this method, the 3D object is constructed additively, layer by layer, according to the pattern defined by a digital micro-mirror device (DMD) as a dynamic mask generator. PµSL 3D printing is a promising high-resolution 3D printing technique in terms of simplicity, accuracy, fast production time, cost-effectiveness, and the variety of materials that it uses. The schematic illustration of the bottom-up configuration^48^ PµSL 3D printers and the 3D CAD model of the designed PµSL 3D printer is shown in Fig. 2.

According to Fig. 2, the fabricated 3D printer consists of a resin tank, a digital light processing engine, a build plate, and a motorized linear translation stage. The digital light processing engine is located under the resin tank and is controlled by the manual linear translation stage to align the projected UV light pattern on the transparent bottom of the resin tank.

The bottom of the resin tank is a UV-transparent and non-stick Teflon film. The build plate is also attached to the motorized linear translation stage and is located above the resin tank. The 3D object is printed hanging from the movable build plate (bottom-up configuration). In this configuration, each newly cured layer is sandwiched between the previous layer and the resin tank. Using DMD allows the photopolymerization of each layer to occur at the same time, and the cured layer is built with a single exposure. With this technology, the printing time is significantly reduced.

In PμSL systems, there is a linear relationship between the lateral accuracy of printing and the maximum lateral dimensions of construction. In this work, to produce the conical microwell mold, the system was set such that lateral accuracy of print in the range of 6 µm with the maximum lateral dimensions (XY build size) of 7.7 mm × 4.8 mm can be achieved. Also, the vertical accuracy of printing (layer thickness) was set in the range of 5 µm. The printing time of the conical microwell mold was about 40 min.

The resin is composed of a photoinitiator (Irgacure 819), an acrylate-based commercial monomer (HDDA), and Sudan I (an absorber which adjusts the light penetration depth). The resins were prepared by mixing 2.6% (w/w) Irgacure 819 in HDDA along with 0.25% (w/w) of Sudan I. The mixtures were sonicated in the dark for 40 min at 40 °C. Then, the stock solutions were kept in a dark place before being used. For removing the non-polymerized resin after printing, the printed objects were washed with isopropanol and dried under a stream of nitrogen. The dried micro-structures were post-cured in 405 nm UV light for 900 s, in the post-curing chamber.

Since PDMS does not fully cure at the surface of the 3D-printed mold made of most commercial acrylate resin^7,49^, the surfaces must be treated with a PDMS compatible material before casting the PDMS. After cleaning, drying, and post-curing, the 3D-printed molds were coated with a PMMA/chloroform solution. The 3D-printed molds were then used in the master mold to replicate the microwell structures in PDMS. PMMA/chloroform solution was made by dissolving PMMA powder at a concentration of 1% w/w in chloroform. The surface of the printed mold was treated with the PMMA/chloroform solution for 10 min at room temperature. Next, the mold was washed with isopropanol and was dried under nitrogen gas.

The geometry, size, and quality of constructions of 3D-printed molds were evaluated by using a Scanning Electron Microscope (FEI-Quanta 200). Before SEM imaging, all samples were fixed on a sample holder and coated with a gold thin film layer for 120 s.

### CNC micro-milling

In this work, the micro-milling process was employed to create microchannels and the main body of the master mold. CNC milled parts of the mold were designed using the SOLIDWORKS 2016 software to create computer-aided design (CAD) files. The SolidCAM 2016 software was employed as Computer-aided manufacturing (CAM) program to convert the CAD files into numerical control (NC) programming language (G-code file) for running the CNC micro-milling machine. Polymethyl methacrylate (PMMA) sheets (Best, Taiwan) in the thickness of 2.5 mm were used as the substrates for mold fabrication in the micro-milling process. A modified 3 axis CNC micro-milling was employed for the micro-machining of PMMA sheets. A two-flute carbide square end mill with a diameter of 1 mm was used as a cutting tool during the micro-milling process. The spindle speed was 18,000 rpm, the feed rate was 60 mm/min, and the depth of the cut was 100 μm. Before micro-milling, the protective film covering the PMMA sheet was removed, and the tip of the cutting tool was zeroed on the top surface of the PMMA sheet via a touchpoint sensor. The fabrication process enabled the creation of channels with low surface roughness while no further physical and chemical treatment were used to enhance the surface quality.

### PDMS casting and device assembly

To fabricate the microfluidic device, PDMS and curing agents were mixed in a 10:1 ratio and stirred for 5 min. The mixture was degassed in a vacuum chamber for 30 min and subsequently poured on the master mold and chip cover mold. Following, the PDMS filled molds were then transferred to an oven at 60 °C for 2 h. The PDMS layers were carefully peeled off from the mold. Based on the method applied to modify the surface of the 3D printed mold, after curing, PDMS can be easily peeled off from the mold. Therefore, the mold can be reused several times without an additional cleaning process. In the next step, the inlet and the outlet holes were punched in the chip cover layer using a 1.25 mm Biopsy punch. Then, chips were flushed with nitrogen gas to clean the channel surface. Afterward, both layers were treated with O_2_ plasma for 120 s and then bound to each other. Finally, the chip cured 3 h at 80 °C in the oven.

### Cell preparation

Human glioblastoma U-87 MG cell line was obtained from the National Cell Bank of Iran (NCBI) affiliated to Pasteur Institute (Tehran, Iran). The cells were routinely cultured in Dulbecco's modified Eagle's medium (DMEM) supplemented with 10% FBS, 1% penicillin–streptomycin at 37 °C in a cell culture incubator at 5% CO^2^, and 95% humidity. Before use, the cells were harvested by trypsinization with 0.25% trypsin at 37 °C. Trypsinization was stopped upon the addition of fresh supplemented DMEM, and cell suspensions were centrifuged at 1000 rpm for 3 min. The cell suspensions were diluted with the cell medium to prepare desired concentrations and seeded into the microfluidic device.

### Cell loading and spheroid formation

Fabricated microfluidic devices were sterilized using a collimated high-power mercury UV light for 1 h. Just before use, the devices were placed in a vacuum chamber for 30 min to prevent the formation of air bubbles. Then, BSA 3% (w/v) was flushed through the microchannels to avoid cell adhesion and incubated for 1 h. In the next step, the chip was rinsed three times with PBS to remove uncoated BSA. The freshly sanitized microfluidic chip was first filled in inlet hole with 100 μL of media through and later gently loaded with cells (1.5 × 10^6^ cells/mL) through the cell inlet using a pipette. After 15 min, fresh medium gently infuses in the inlet, and the waste medium containing Excess cells that did not enter microwells was removed from the chip. After cell seeding, the microfluidic device was incubated to allow spheroid formation. The time of the cell loading and fresh culture medium change is 60 s. The culture medium was exchanged once a day to ensure sufficient nutrient supply to the cells. Spheroid growth tracking overtime was also examined by everyday imaging^50^. Bright-field and epi-fluorescence imaging were conducted on a Zeiss Axiovert 40 CFL inverted routine microscope. The spheroid diameter was measured by using Fiji open-source software^51^. For evaluation of the cell viability, spheroids were stained with the fluorescent dyes AO (5 mg/mL), and PI (5 mg/mL) in PBS solution for 5 min and then the images were taken.

## Results and discussion

### Fabrication of the microfluidic device

**Figure 3:**
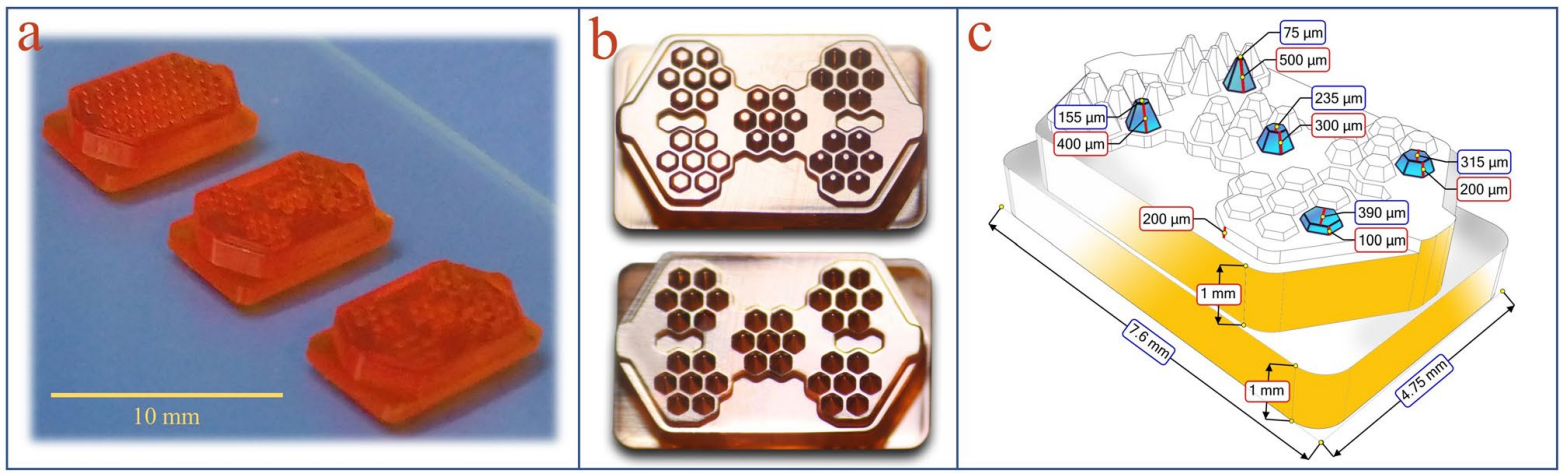
(**a**) Three types of 3D printed molds with different designs. (**b**) Micrographs of the 3D printed molds with the microwells of variable heights and constant height. (**c**) Details of designing the variable height microwell mold.

Microwell arrays and microwell chips are widely used as efficient methods for the production of the 3D tumor spheroid model. Various groups have shown that conical or concave microwells are more efficient in producing spheroids than flat-bottomed microwells. The conical shape of the microwell bottom contributes to better cell aggregation^27,29,32,33,42^. Different methods have been used for fabricating the non-flat-bottomed microwell chips. For example, Liu et al. used an ice lithography method carried out on superhydrophobic substrates to fabricate a quasi-spherical microwell chip^52^. Lee et al. also made a concave microwell chip using a double PDMS casting procedure from the PMMA mold that was made by a laser carving machine^53^. In a creative and simple method, Kim et al. fabricated a concave-bottomed microwell chip using the capillary action of liquid polymer on the pins of a computer CPU, which is achieved without expensive materials or complicated procedures^54^. The main problem with such methods is the lack of flexibility to create an object with different shapes in different sizes. In an alternative approach, commercially available additive manufacturing tools such as SLA printer were applied^55^. The low resolution of these devices is a significant problem for the fabrication of the conical or concave microwells. The minimum accuracy of construction required for this application is 10 µm, while the resolution of commercial machines is at best 50 micrometers^4^. In this study, the lab-made PµSL 3D printer with 6 µm lateral resolution was used to make the microwell mold. Figure 3 illustrates the printed microwell molds. The microwells can be designed with different 3D structures, which highlighted the flexibility of the proposed method. For example, Fig. 3a shows the three types of 3D printed molds with different designs. Figure 3b depicts the micrographs of the 3D printed mold with the microwells of variable heights and constant height. For increasing mechanical strength, the printed molds were re-irradiated in a UV chamber. Figure 3c shows the details of designing the variable height microwell mold.

**Figure 4:**
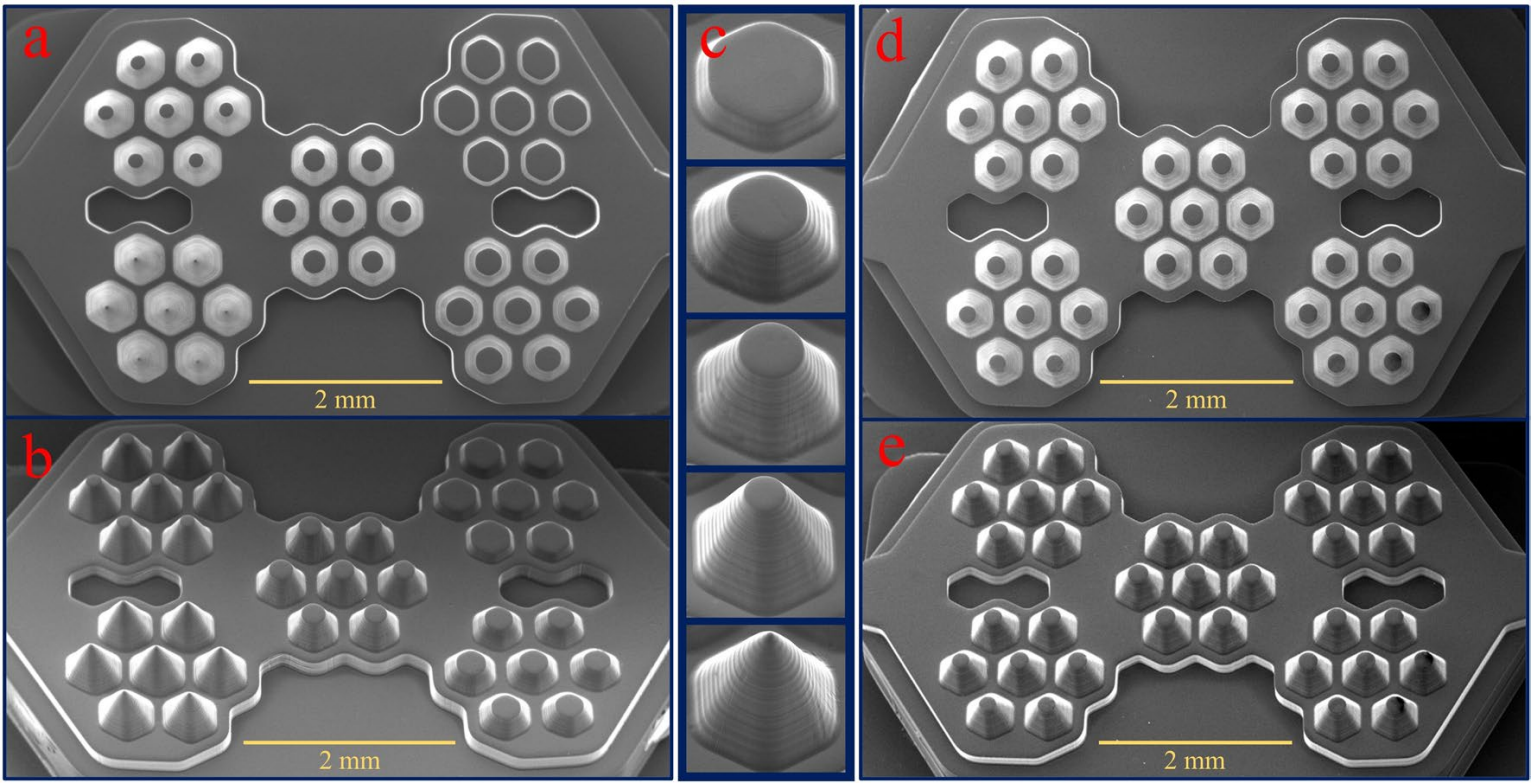
Scanning electron micrographs of 3D printed molds: (**a**, **b**) Top and side view of the mold with variable microwell heights. (**c**) Side view of the mold for different microwells from 100 to 500 µm. (**d**, **e**) Top and side view of the mold with constant microwell heights (300 µm).

According to the flexibility of design, we examined the influence of microwell depth on spheroid formation in a single chip. Figure 4 shows the scanning electron micrographs of 3D printed molds. In Fig. 4a,b, the top and side view of the mold with variable microwell heights have been shown. In Fig. 4c the mold for different microwells from 100 to 500 µm have been depicted. The angles and dimensions of the printed objects precisely correspond to the target designs. Also, due to the high vertical resolution of construction, surface smoothness is appropriate for molding applications. SEM micrographs of top and side views of the mold with constant microwell height (300 µm) have been shown in Fig. 4d,e.

**Figure 5:**
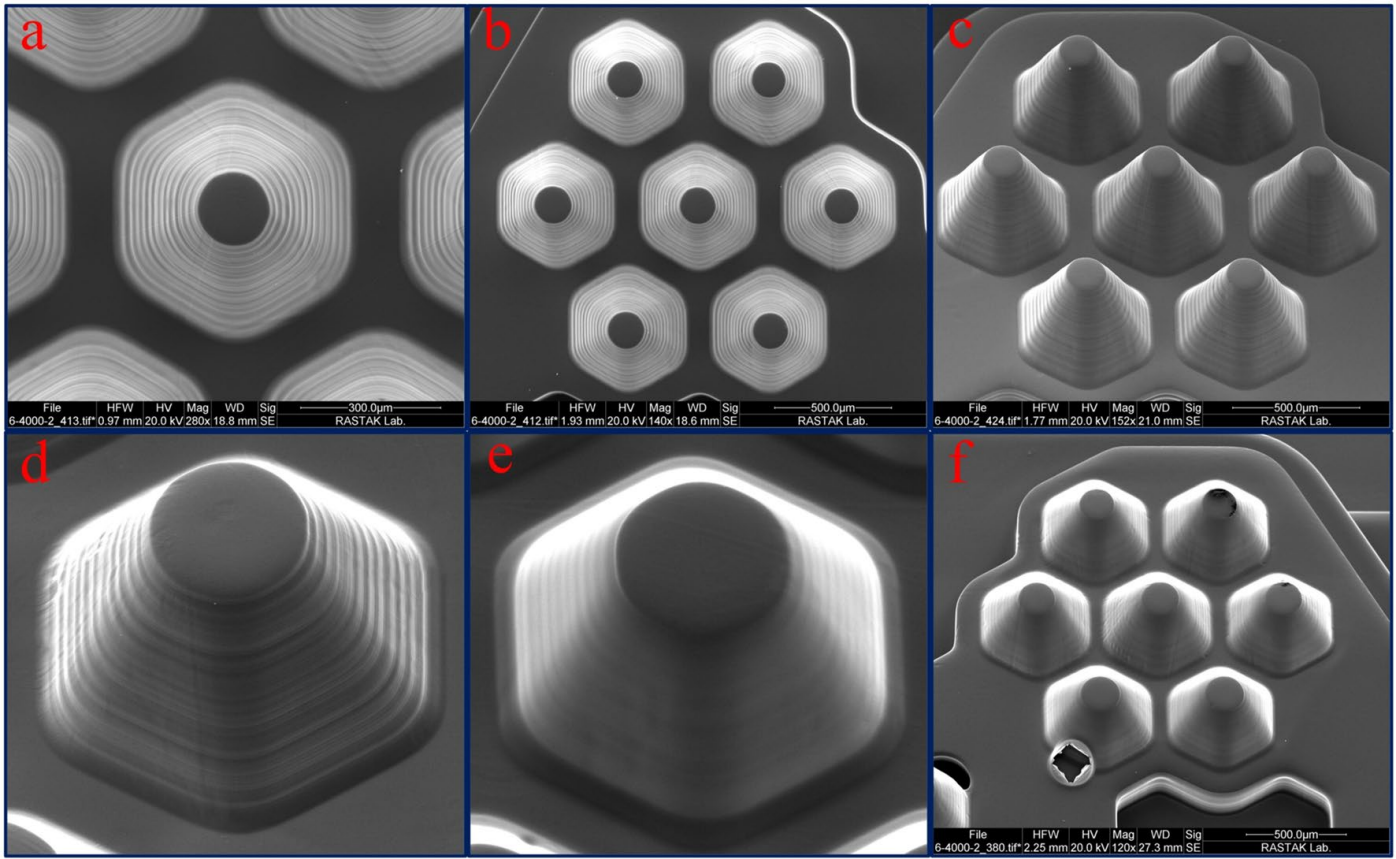
Scanning electron micrographs of the microwell molds. (**a**) Top view of a single 400 µm microwell mold. (**b**, **c**) Top and side views of microwells with a height of 400 µm. (**d**, **e**) Side view of a 300 µm microwell before and after PMMA coating. (**f**) An example of the failure in the PMMA layer causes by bubble formation.

The previous study has shown that by engineering and optimizing the optical system of PµSL 3D printer and adjusting the pattern of ultraviolet radiation on the photopolymer surface, the intensity of the exposed light can be perfectly uniform, which leads to the different parts of the surface are exposed to the same light intensity^16^. As a result, the thickness of the layer created is the same over the entire surface. In the present study, it is particularly important because the shape of a microwell is repeated several times. Figure 5 shows the reproducibility of the system in the 3D printed mold. Figure 5a shows the symmetry for one 400 µm microwell mold with large magnification. In Fig. 5b,c, the SEM micrographs of top and side views of microwells with a height of 400 µm, have been shown. It is pretty clear that the mold of all printed microwells is similar. The best vertical accuracy of this 3D printer is 1 µm, as shown in a previous report^16^, but the accuracy required for this study is 5 µm. for this reason, the vertical accuracy was adjusted to the value of 5 µm.

Various resin components prevent PDMS polymerization on the contact surface, which causes the PDMS layer to adhere to the mold surface after curing. For solving this problem, a PMMA/chloroform solution was used for coating the printed mold surface. To evaluate the thickness of the PMMA layer formed on the 3D printed mold, the surface of a glass slide was covered by this solution. Then the thickness of the formed layer was measured by a Dektak XT (Bruker Corporation) profilometer. The measured thickness of the PMMA layer was about 10 µm.

There are several advantages to using a PMMA/chloroform solution before the PDMS casting process. In this method, a layer of PMMA is coated on the 3D printed parts. This layer increases the surface's smoothness and prevents the PDMS layer from adhering to the mold surface after curing. Due to the good mechanical strength and high chemical stability of PMMA, the mold coated with this method can also be used several times without impairing the quality of the PDMS casting and curing process. For example, in this work, we used each master mold at least 25 times. Furthermore, chloroform is an appropriate solvent for PMMA and has a high evaporation rate, which helps create a uniform layer of PMMA on the printed parts. Besides, the chloroform solvent does not damage the 3D printed part.

The coating performance on the mold surface is shown in the scanning electron micrographs (Fig. 5d,e). Accordingly, after using the PMMA, a uniform layer is formed on the mold surface. Due to the three-dimensional nature of the mold, it is necessary to pour the solution slowly and in one step over the surface so that no bubbles are formed on the surface. Figure 5f shows the failure of the PMMA layer causes by bubble formation. However, some new resins such as TF-3D have also been introduced and can be used to perform PDMS casting on printed parts without surface modification^56^.

**Figure 6:**
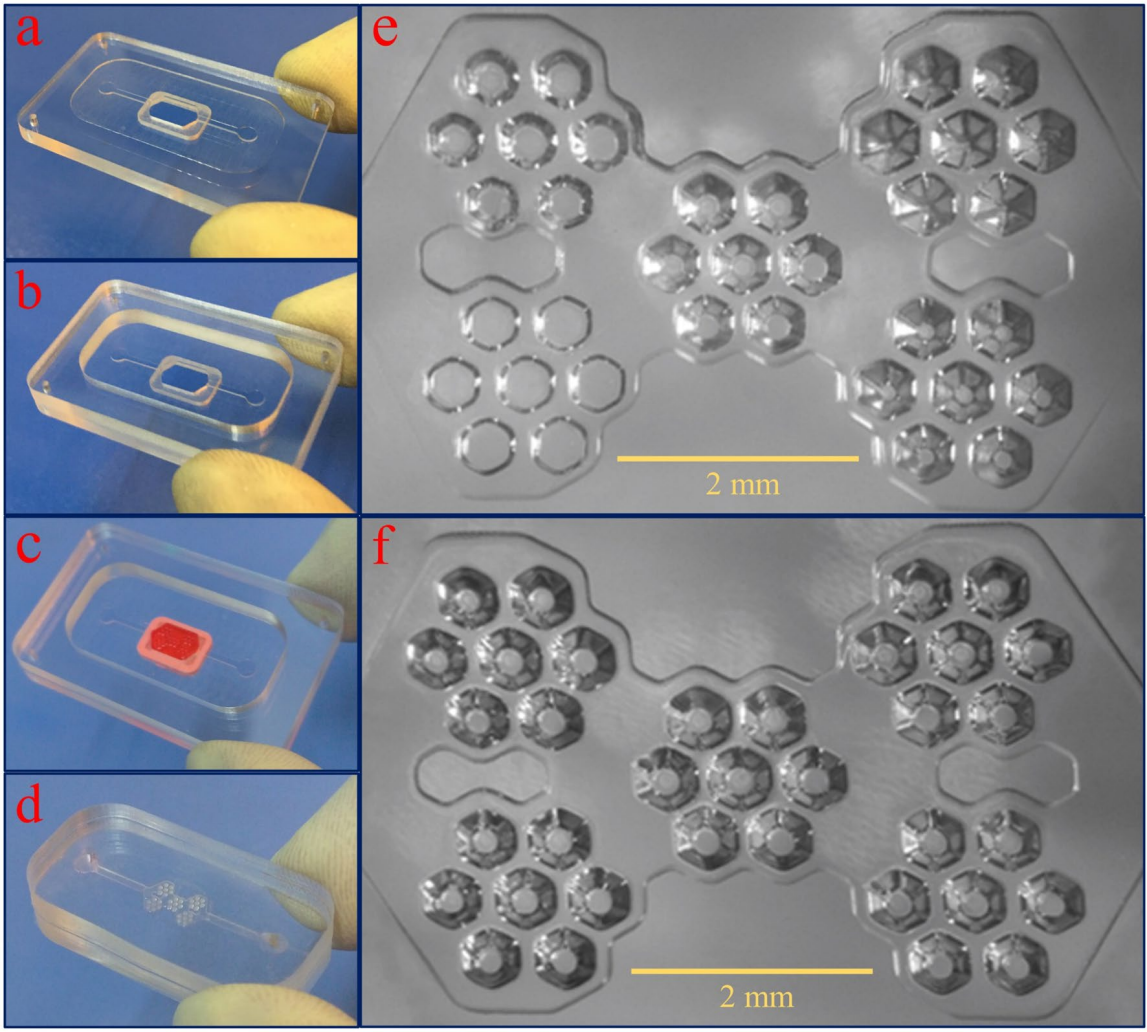
Steps to produce the master mold and casting with PDMS. (**a**, **b**) Two CNC milled parts are bonded to each other permanently using a solvent bonding method. (**c**) The 3D printed mold is assembled on the CNC mold and fixed with reusable putty adhesive. (**d**) The cover and microwell PDMS layers bonded together using the plasma bonding method to produce the microwell chip. (**e**, **f**) The optical micrographs of PDMS layers with the variable and constant depth (300 µm) microwells.

Manufacturing high-resolution microfluidic components using conventional techniques always requires some sophisticated laboratory facilities and skilled person. Therefore, it's necessary to simplify the manufacturing process of a microfluidic chip as much as possible so that it can be used in a biological laboratory. Figure 6 illustrates the steps to produce the master mold and casting with PDMS. In Fig. 6a,b, two CNC milled parts are bonded to each other permanently using a solvent bonding method. In Fig. 6c, the 3D printed mold is assembled on the CNC mold and fixed with reusable putty adhesive. High thermal resistance and the ability to detach from the master mold are the advantages of using this glue. For producing the chip, the cover and microwell PDMS layers bonded together using the plasma bonding method (Fig. 6d). Figure 6e,f show the optical micrographs of PDMS layers with the variable and constant depth microwells.

### Flow simulation

**Figure 7:**
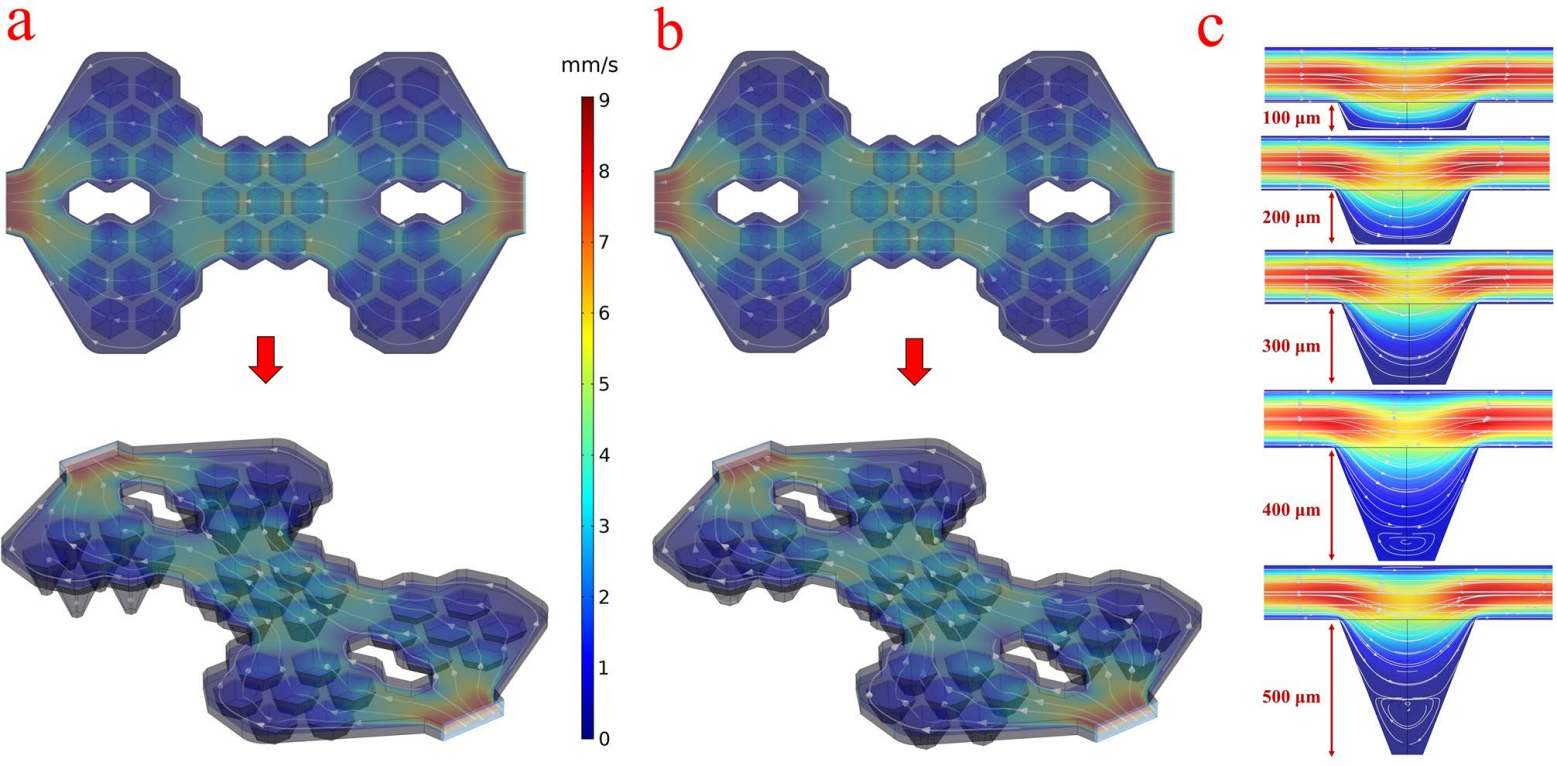
Numerical fluid flow simulation. (**a**, **b**) The velocity distribution and streamline of the fluid flow inside the chamber and microwells for two chips with variable depth microwells and 300 μm depth microwells. (**c**) The cross-section of the velocity distribution and streamline of the fluid flow inside the microwells. [This figure was created by COMSOL 5.1 (www.comsol.com)].

Figure 7a,b shows the result of numerical fluid flow simulation for two chips with variable depth microwells and constant depth (300 μm) microwells. Accordingly, for each group of seven microwells, the flow distribution is uniform, and there is no significant difference between the two chips. It seems that the depth of the microwells does not affect the main flow of chips, and therefore it can be assumed that the conditions are the same for each group of microwells. This simulation furthermore showed that the flow rate in the microwell is less than the upper layers, which helps cell trapping and spheroid formation. Figure 7c shows the cross-section of the microwells, and it turns out that with increasing depth of the microwell, the flow rate decreases, and as a result, the cell aggregation and spheroid formation are facilitated. But at a depth of greater than 300 μm, in the microwell, a secondary flow is generated. The flow profile inside the chip and secondary flow in the deep wells depends on the chip geometry and does not change significantly with the flow rate changes. According to the simulation results, the flow profiles for the two flow rates, 50 µL/min (rate to cells loading) and 30 µL/min (rate to remove unseeded cells) are similar. In microwells with a depth of more than 300 μm, secondary flow is generated at any flow rate. The Supplementary Movie S1 shows that secondary flow is also generated with a low flow rate (from 500 to 5 µL/min) in microwells with a depth of 500 μm.

Secondary flow prevents the escape of cells from the microwells, but at the same time limits the exchange of nutrients, oxygen, and excretory materials between the cells and the fluid flow. Finally, the 300 μm, appears to be a suitable depth for the formation and maintenance of a tumor spheroid.

### 3D-spheroid formation and characterization of spheroids

**Figure 8:**
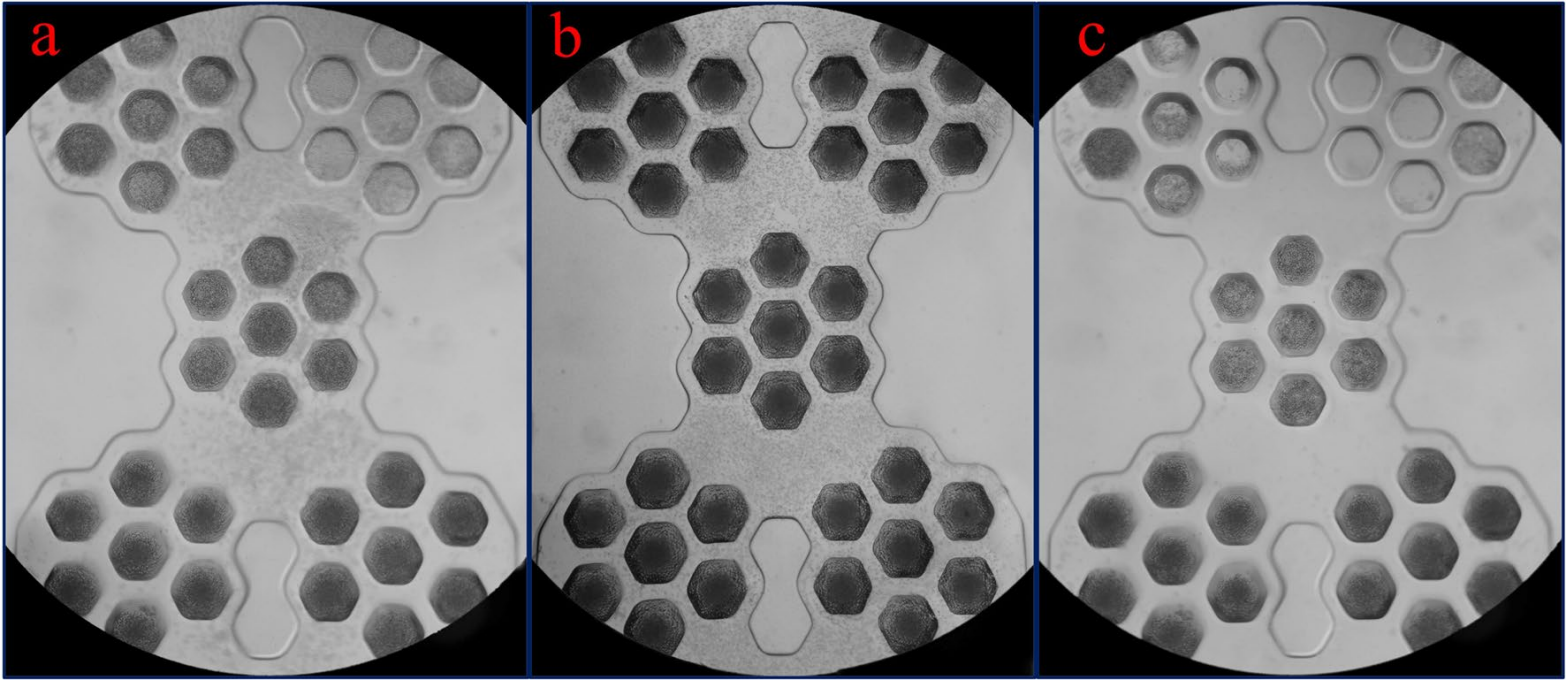
(**a**) The chip with variable depth microwells after cell injection. The difference in the depth of the microwells significantly influences for trapping cells. (**b**) The chip with constant depth (300 µm) microwells after cell injection. The density of trapped cells is uniform for all microwells. (**c**) Microwells after washing with the culture medium. Some microwells with a depth of 100 and 200 μm were emptied after washing.

We performed experiments to validate the formation of the tumor spheroids in the microwells by seeding U-87 MG cells into the microfluidic chip. Cells can be inserted into the chip using both a syringe pump and pipetting, which reflects the ease of applying this chip. The solution a 1.5 million cells/mL was injected with a syringe pump at a flow rate of 50 µL/min. At this rate, the microwells are filled with cells after 60 s, which reduces cell loss. After 15 min, 100 μL culture medium was injected into the chip at the flow rate of 30 µL/min to remove the unseeded cells from the chip. Figure 8a,b show the chips with variable depth and constant depth (300 µm) microwells, respectively, after cell injection. The difference in the depth of the microwells significantly influences for capturing cells, which is clearly shown in Fig. 8a for chip 1. while in chip 2, the density of trapped cells is uniform for all microwells (Fig. 8b). It appears that, as the microwell depth increases, the number of trapped cells increases, and the probability of the cell escaping due to the fluid flow decreases. Figure 8c shows the microwells after washing with the culture medium. Some microwells with a depth of 100 and 200 μm were emptied after washing, while cells remained in microwells deeper than 300 μm.

Increasing the depth of the microwell reduces the shear stress of the fluid flow to the cells and prevents the spheroid inside the microwell from directly exposing to the fluid flow. However, it is difficult to diffuse nutrients, respiratory gas, and waste into or out from deep microwells. Also, in deep microwells, spheroids can be located at different distances from the microwell surface, so not all microwells can be imaged simultaneously. In this case, the focal position of the microscope must be adjusted separately for each microwell.

**Figure 9:**
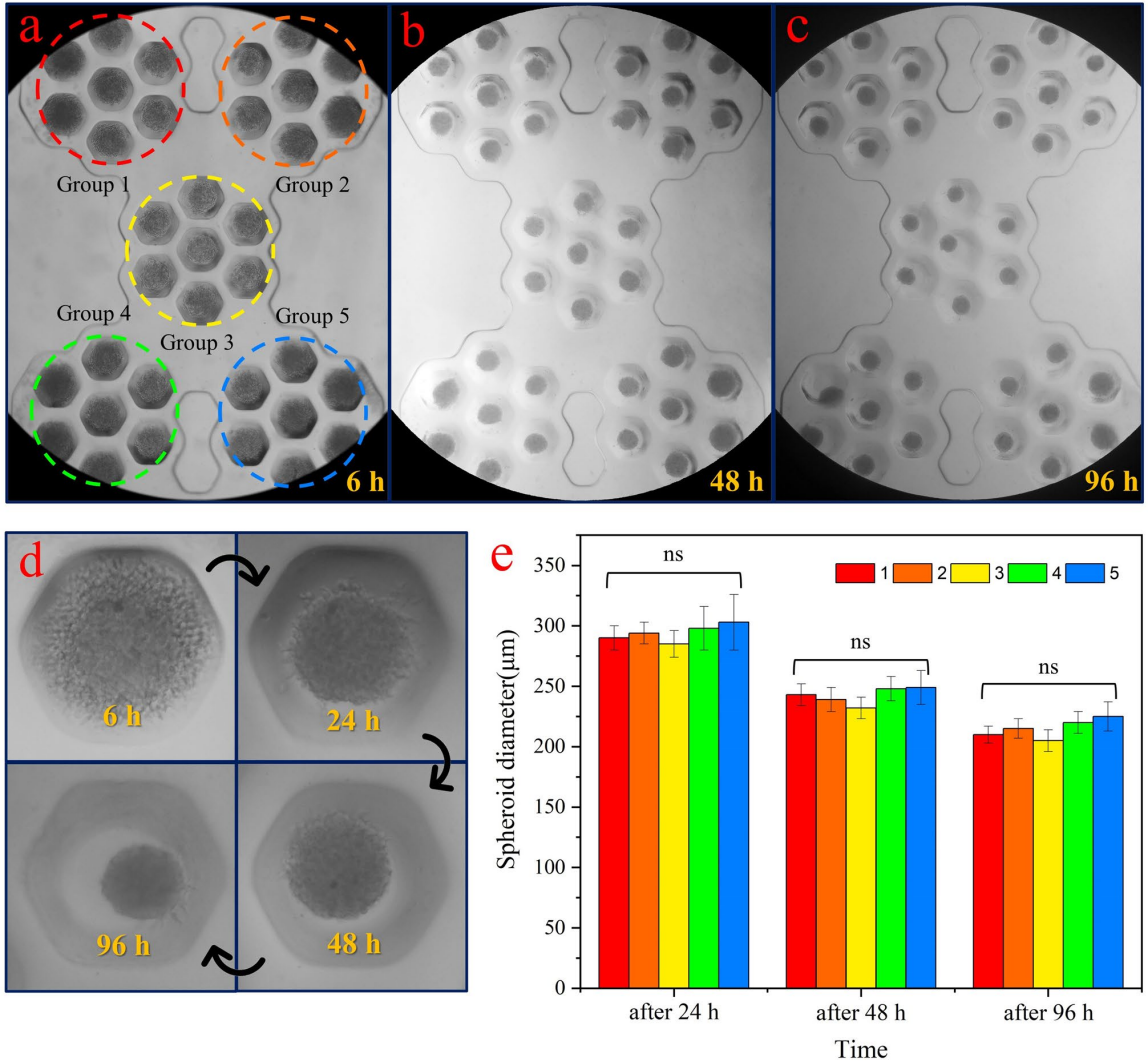
(**a**–**c**) The formation of a spheroid in microwells with a depth of 300 μm after 6 h, 48 h, and 96 h. In all microwells, the spheroid is formed. (**d**) Increment of the compactness of the spheroid for four days in a single microwell. (**c**) The diameter of the cultured spheroids for each group versus time. There is no significant difference in the diameter (ns) of the spheroid between these five groups of microwells.

Based on the experimental and numerical results, the depth of 300 μm was selected as the optimal microwell depth to generate the spheroid. Figure 9a–c show the formation of a spheroid in microwells with a depth of 300 μm after 6 h, 48 h, and 96 h. As clearly shown, in all microwells, the spheroid is formed. The results indicate an increment of compactness of the spheroid for four days, which has been demonstrated in large magnification for a single microwell in Fig. 9d. Since each group of microwells has a particular position than the input and output, the diameter of the cultured spheroids for each group was measured separately. The results are given in Fig. 9e. At least three independent experiments were carried out for each microwell depth. Data were presented as mean ± SEM. One-way ANOVA was carried out to evaluate statistical differences. The p-value of 0.05 was considered significant. As it turns out, there is no significant difference in the diameter of the spheroid between these five groups of microwells.

**Figure 10:**
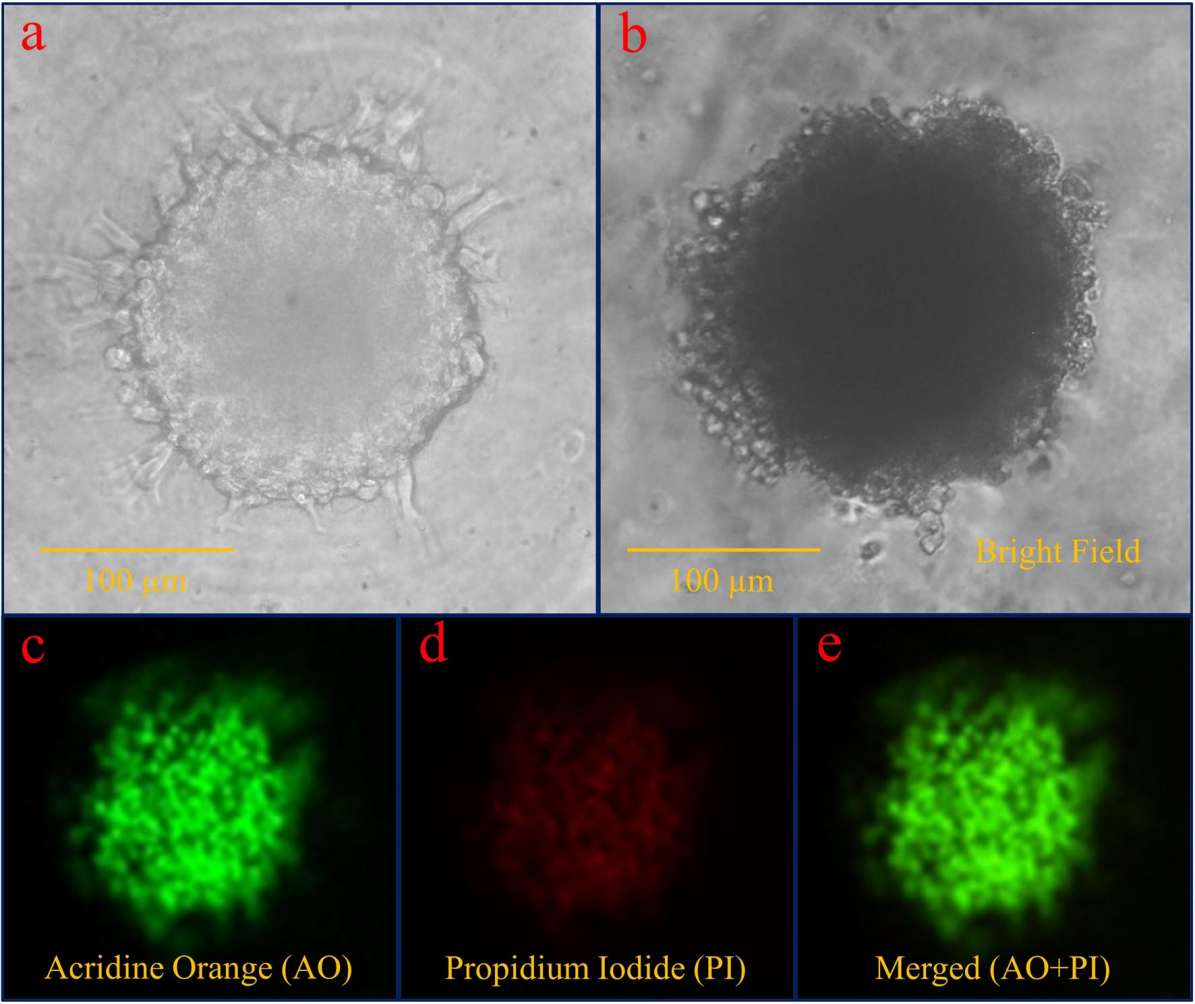
(**a**) The spheroid appears to have taken the shape of the container (hexagonal shape) during cell aggregation. (**b**–**e**) The result of the viability assay using AO/PI staining after 4 days of spheroid formed. Green color for merged image illustrates the high percentage of live cells versus dead cells.

Figure 10a shows a spheroid cultured in the microwell with large magnification. Spheroid-derived cells display an invasion process that is expected by U-87 MG cell type and indicates that the spheroid formed in the microwells can be phenotypically used for various studies. Since the microwell shape is six-sided, the spheroid appears to have taken the shape of a container during cell aggregation. Figure 10b–e shows the result of the viability assay using AO/PI staining after 4 days of spheroid formed. Green color for merged image illustrates the high percentage of live cells versus dead cells. Based on the image analysis, it was estimated that the viability of the cells of the proliferating layer was over 90%.

## Conclusions

In the present study, we developed a new method for fabrication of a large size master mold containing high-resolution 3D microstructures. Manufacturing of high-resolution and low-resolution components was carried out using the lab-made PµSL 3D printer and the CNC micro-milling methods, respectively. In the next step, fabricated components assembled to form a master mold, which is employed for PDMS casting. To evaluate this approach, we used it to make a conical microwell-based microfluidic chip for generating tumor spheroid. The microwell depth directly affects the shear stress of fluid flow and diffusion of the reagents. Accordingly, to investigate the effect of depth on the spheroid formation, we developed chips with various microwell depths. Experimental results showed, as the depth of the microwell increases, the number of cells captured in the microwell increases, and the probability of the cell escaping due to fluid flow decreases. But at a depth of greater than 300 μm, the numerical simulation results indicated that a secondary flow is generated in the microwell. Secondary flow prevents the escape of cells from the microwells, but at the same time limits the exchange of nutrients, oxygen, and excretory materials between the cells and the fluid flow. Also, in deep microwells, spheroids can be located at different distances from the microwell bottom, so not all microwells can be imaged simultaneously. In this case, the focal position of the microscope must be adjusted separately for each microwell. According to experimental and numerical results, a depth of 300 μm was selected as the optimal microwell depth to generate the spheroid. The results exhibited that at a depth of 300 μm, this platform enabled tumor spheroids culture with uniform structure. Ultimately, using the established method, it is possible to construct the desired microfluid high-resolution chip with customized size. Furthermore, the developed microwell chip is a promising tool for the practical formation of spheroids in biological laboratories to evaluate anticancer drugs' effectiveness.

## Supplementary Information

Supplementary Video S1.
