# Supplementary figures and images for: A combined 3D printing/CNC micro-milling method to fabricate a large-scale microfluidic device with the small size 3D architectures: an application for tumor spheroid production
Source: Sci Rep. 2020 Dec 17;10:22171. doi: 10.1038/s41598-020-79015-5 (PMC7747638; doi:10.1038/s41598-020-79015-5)

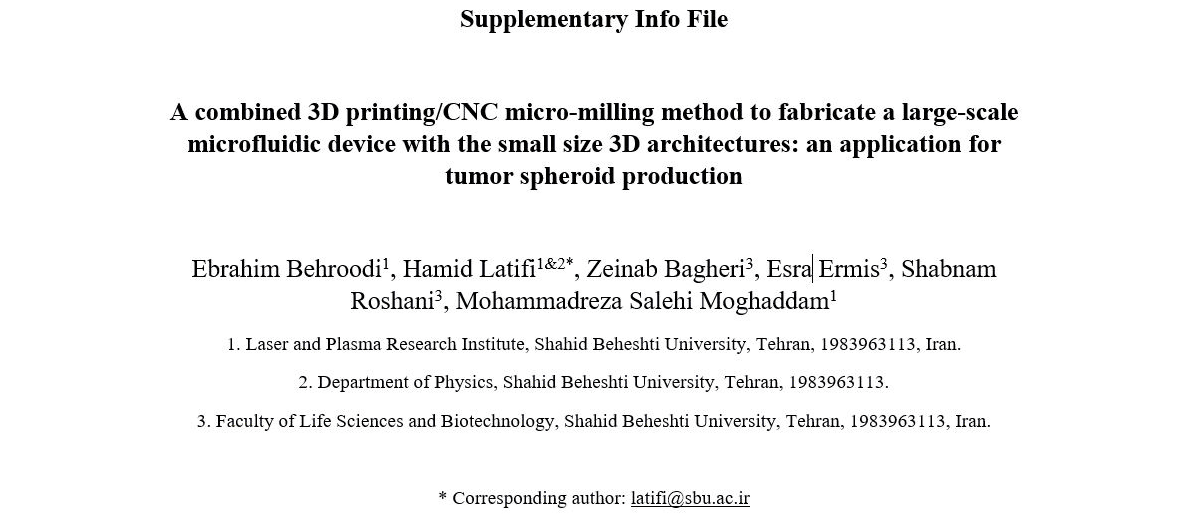

Supplement: Supplementary file 1 — Supplementary Video S1. [file 41598_2020_79015_MOESM1_ESM.gif]
